# Supplementary figures and images for: A serpin gene from a parasitoid wasp disrupts host immunity and exhibits adaptive alternative splicing
Source: PLoS Pathog. 2023 Sep 11;19(9):e1011649. doi: 10.1371/journal.ppat.1011649 (PMC10513286; doi:10.1371/journal.ppat.1011649)

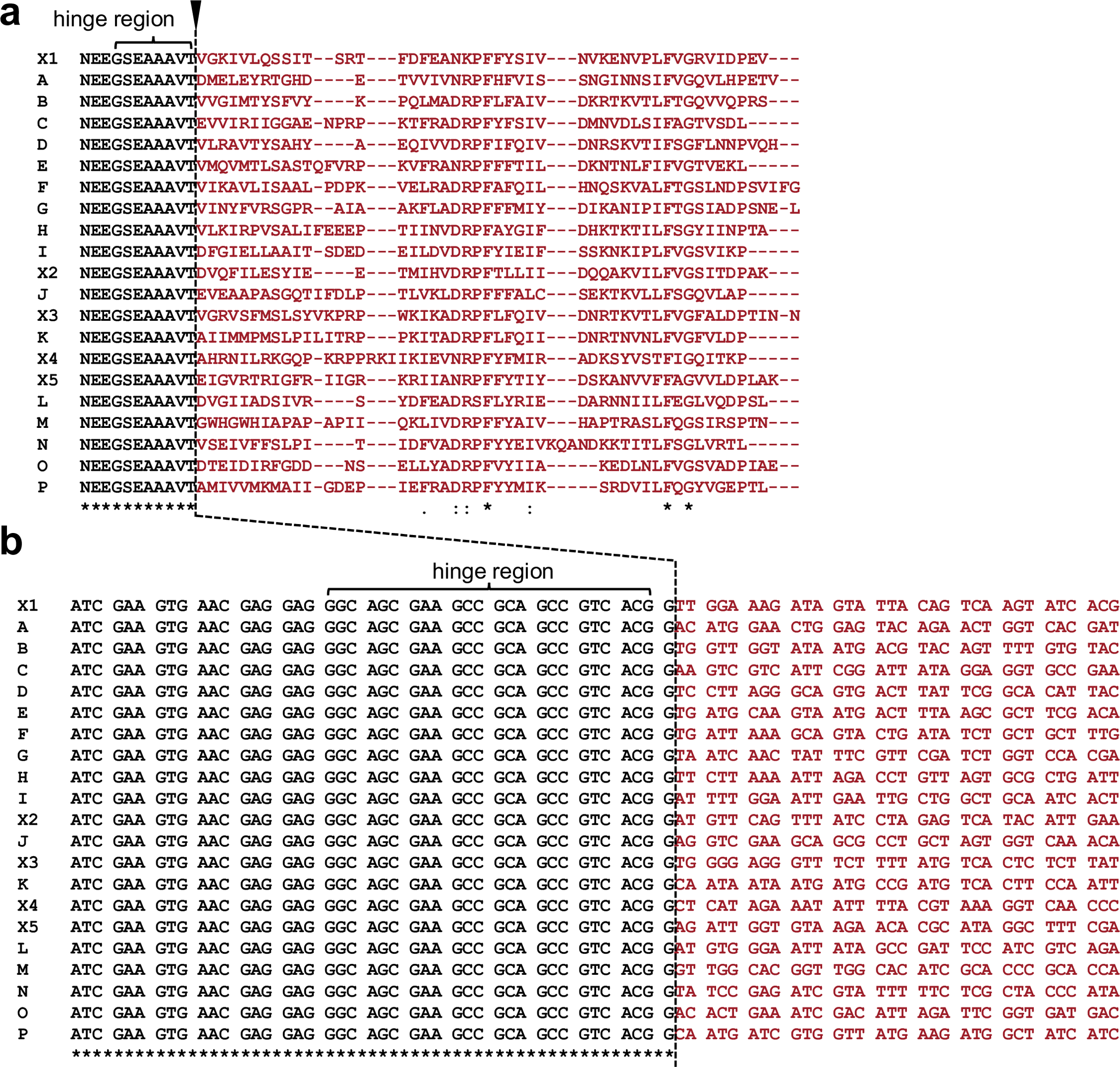

Supplement: S1 Fig — The black arrow indicates the splicing site. Curly brackets indicate the hinge region. (TIF) [file ppat.1011649.s005.tif]

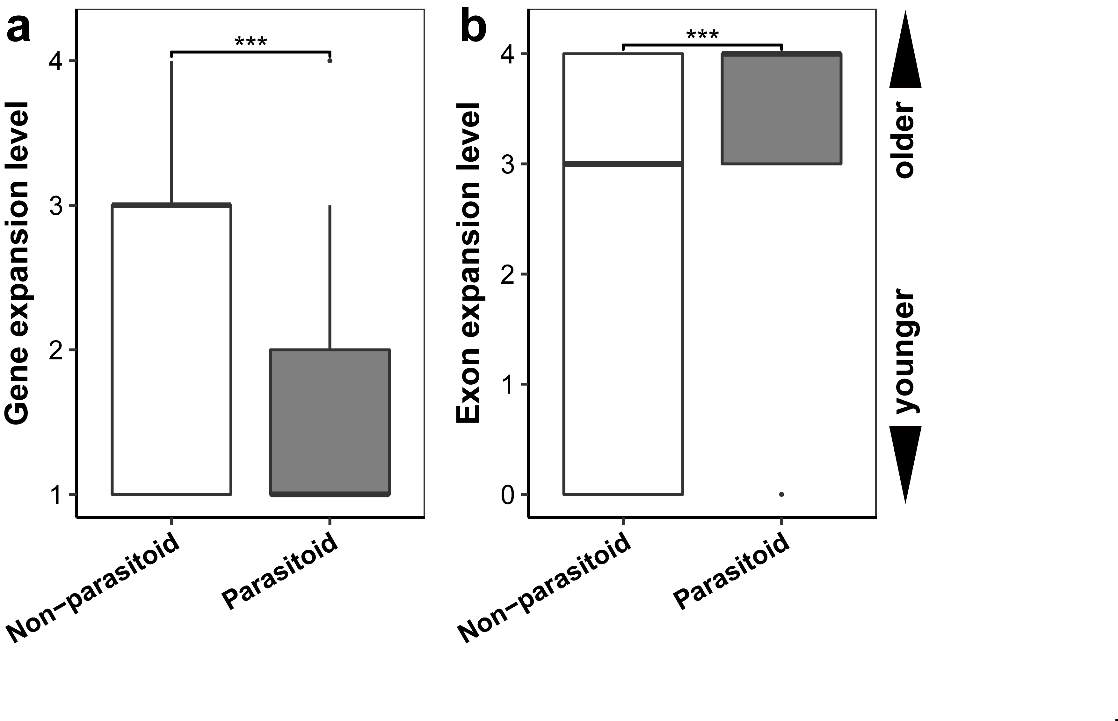

Supplement: S3 Fig — Expansion levels were compared using a ranking method. 0 indicates gene-specific expansion; 1 indicates genus-specific expansion; 2 indicates family-specific expansion; 3 indicates order-specific expansion; 4 indicates not specific. Smaller rank numbers mean younger expansions. (TIF) [file ppat.1011649.s007.tif]

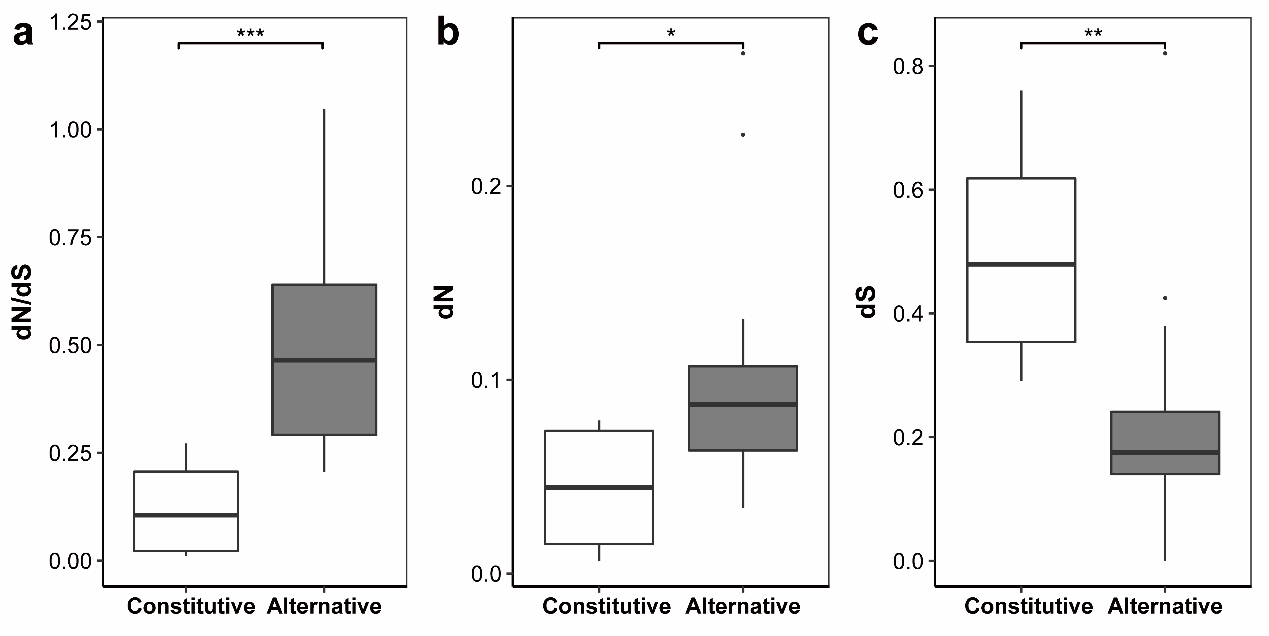

Supplement: S4 Fig — Constitutive exons include exons 2–7, and alternative exons include 8B, 8C, 8D, 8E, 8F, 8G, 8H, 8I, 8J, 8K, 8X4, 8X5, 8L, 8N, 8O, and 8P. (TIF) [file ppat.1011649.s008.tif]

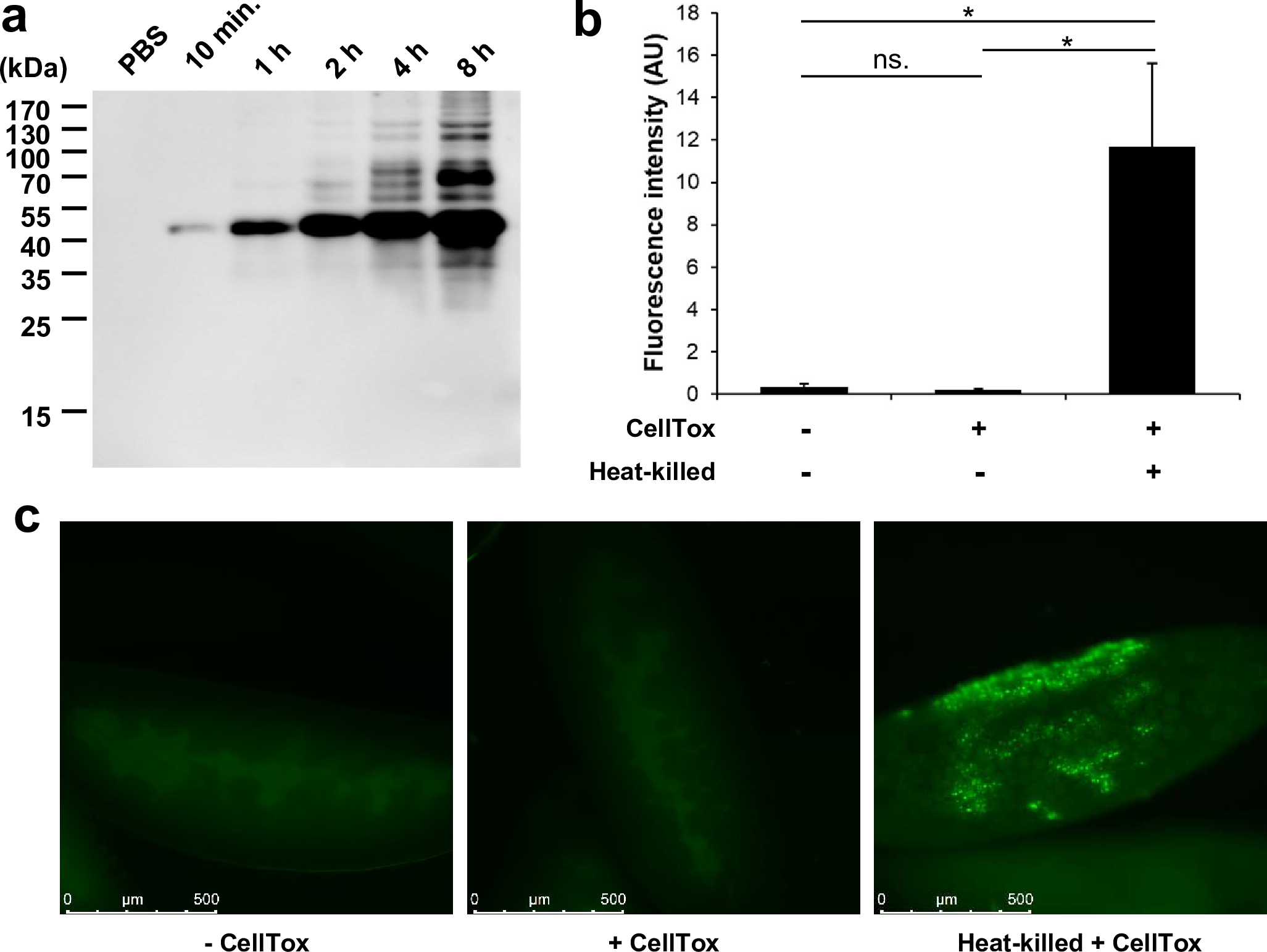

Supplement: S5 Fig — (a) Western blot detection of PpSerpin-1 proteins in PBS incubated with P. puparum larvae. (b) Fluorescence intensity of larvae after 8 h of incubation in PBS. CellTox is a dye that detects cell integrity and can enter ruptured cells and bind to DNA to emit fluorescence. The stronger the fluorescence intensity, the more ruptured cells in the larvae. The larvae remained viable after incubation 8 h, and there was no significant difference compared to the spontaneous fluorescence without CellTox added. Larvae were heat-killed at 100°C for 10 min and used as the positive control. (c) Representative fluorescence images of P. puparum larvae after 8 h of incubation in PBS. * p < 0.05; ns.: p > 0.05. (TIF) [file ppat.1011649.s009.tif]

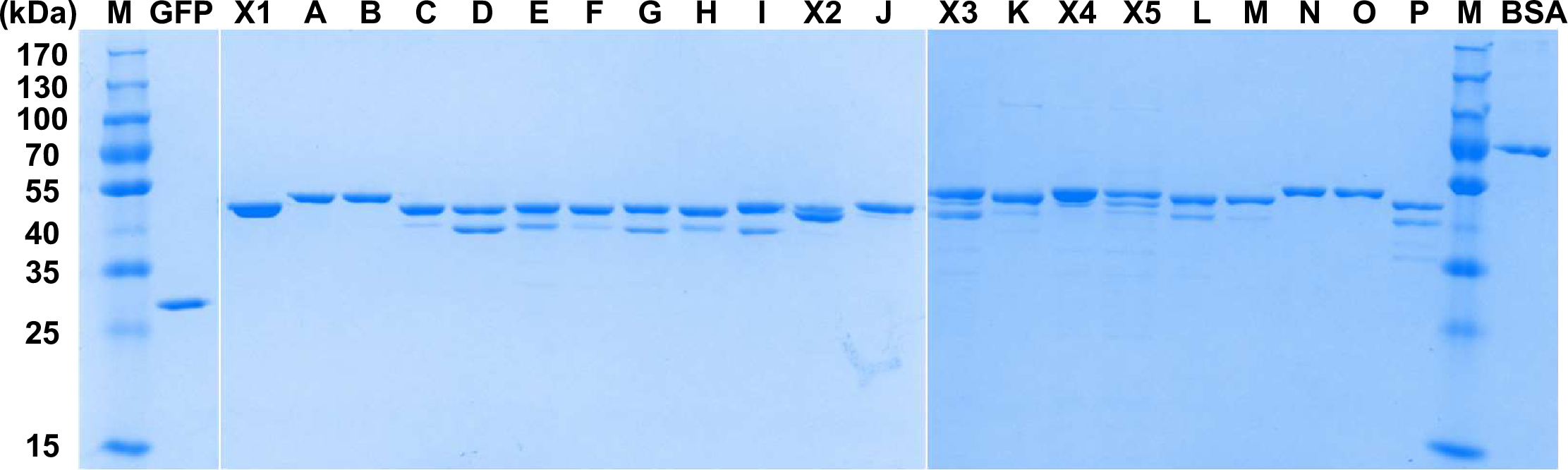

Supplement: S6 Fig — Some isoform proteins show two bands, which might be truncated by-products during the expression process. (TIF) [file ppat.1011649.s010.tif]

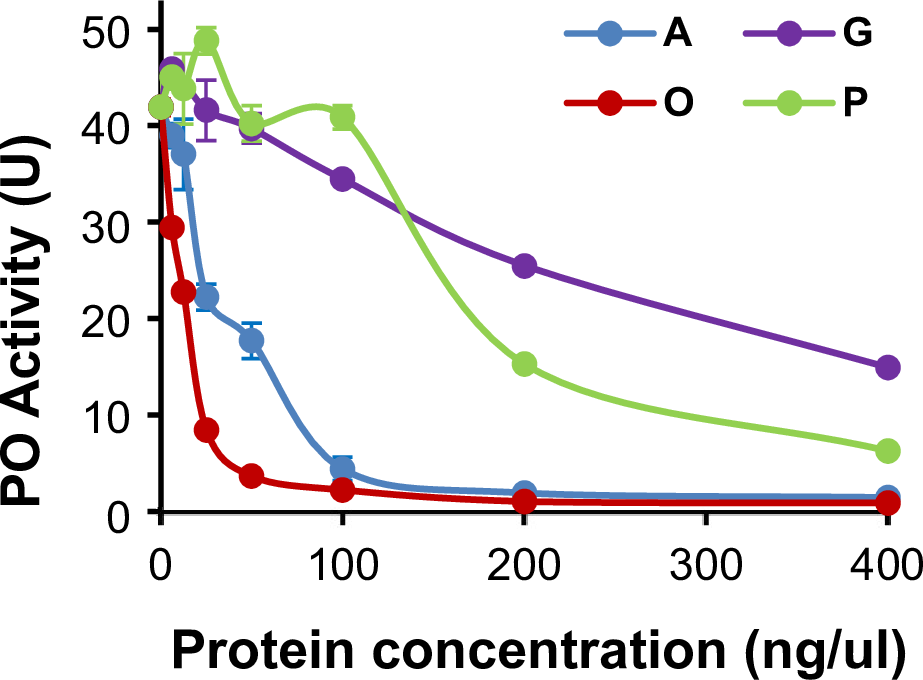

Supplement: S7 Fig — (TIF) [file ppat.1011649.s011.tif]

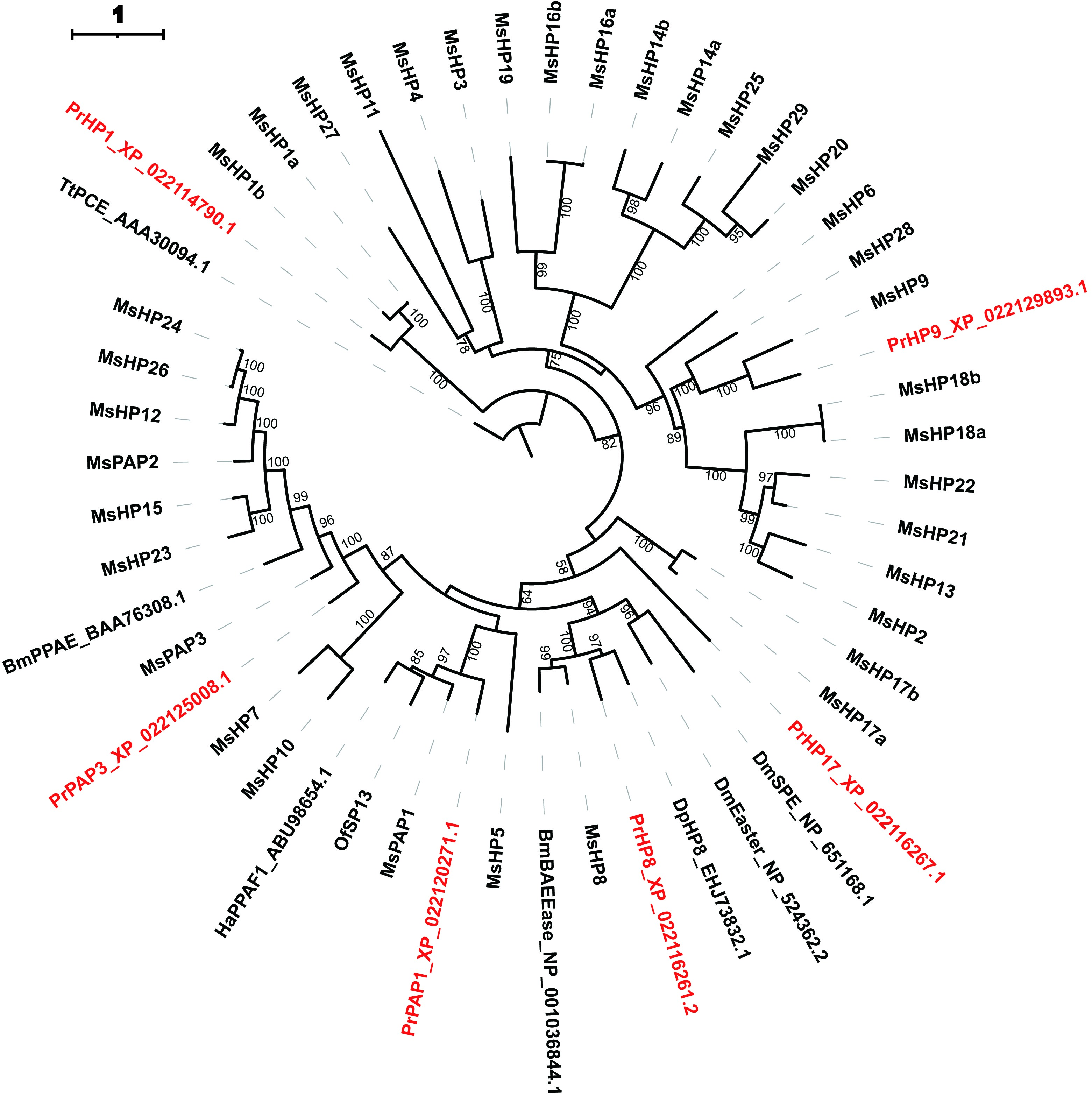

Supplement: S8 Fig — Sequences of MsHP and MsPAP were retrieved from [49] paper. Sequence, alignment and tree files can be downloaded at FigShare (https://doi.org/10.6084/m9.figshare.21545598.v1). (TIF) [file ppat.1011649.s012.tif]

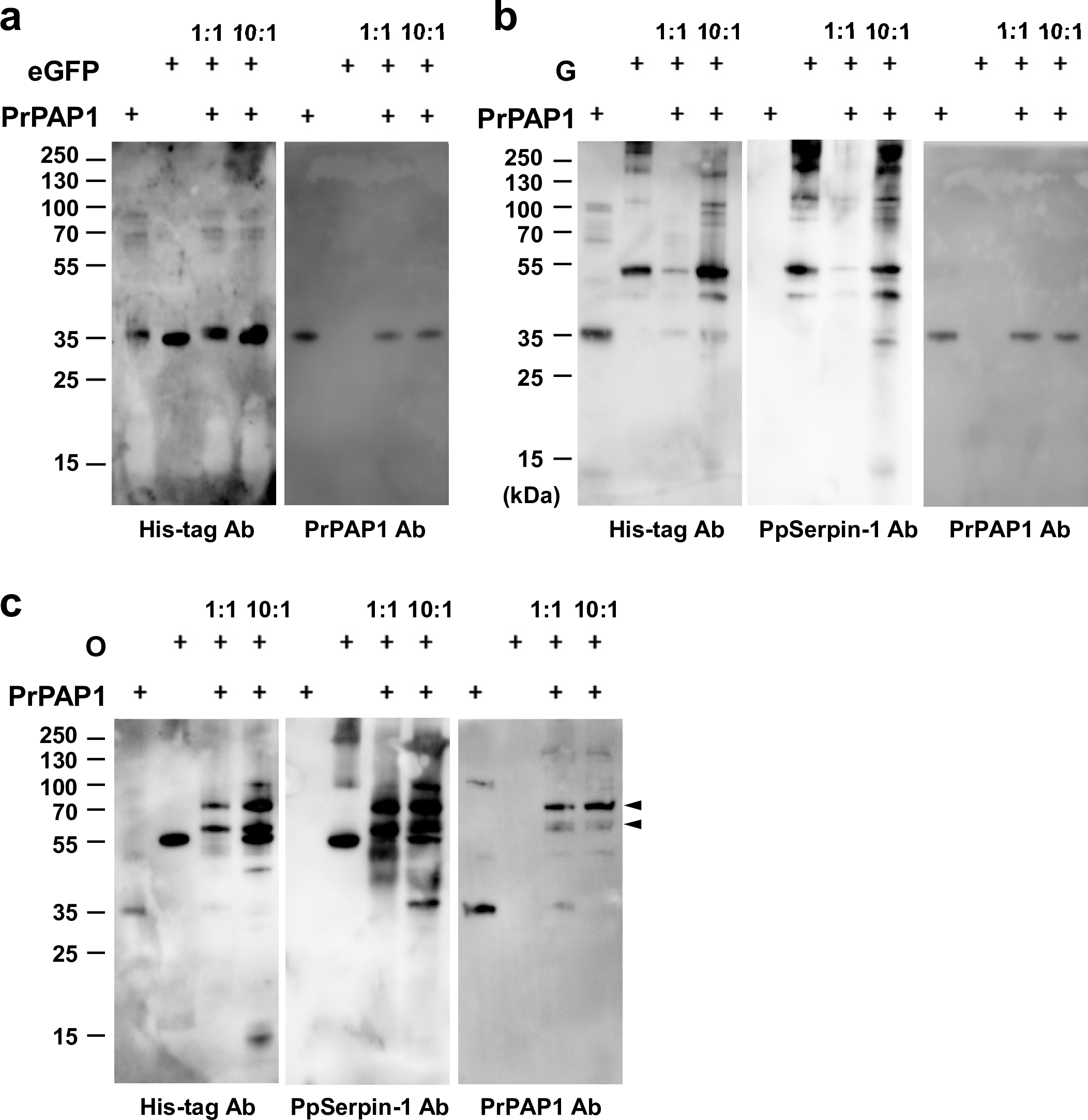

Supplement: S9 Fig — Black arrows indicate complexes formed for PpSerpin1 isoforms with PrPAP1. (TIF) [file ppat.1011649.s013.tif]
