# Supplementary material for: A serpin gene from a parasitoid wasp disrupts host immunity and exhibits adaptive alternative splicing
Source: PLoS Pathog. 2023 Sep 11;19(9):e1011649. doi: 10.1371/journal.ppat.1011649 (PMC10513286; doi:10.1371/journal.ppat.1011649)
Supplement: Attachment — Submitted filename: point-to-point-response letter-20230808.docx [file ppat.1011649.s014.docx]

**Re: Decision letter for PPATHOGENS-D-23-00784**

Dear Dr Jiggins,

Thank you for giving us the opportunity to revise our manuscript “A serpin gene from a parasitoid wasp disrupts host immunity and exhibits adaptive alternative splicing”.

We appreciate the careful review and constructive suggestions for us to improve our manuscript and have taken all the comments into consideration. 1) We conducted additional experiments to confirm that larval saliva of *P. puparum* inhibits host melanization, and this inhibitory effect could be eliminated by antibodies against PpSerpin-1. We also demonstrated that *P. puparum* larvae remained viable and showed no significant rupture after being incubated in PBS for 8 h. 2) We conducted additional Western blotting and detected PpSerpin-1 in the venoms of *P. puparum* and its relative species *N. vitripennis*, *Muscidifurax raptor*, and *M. uniraptor*. By examining published venom sequences, we also identified PpSerpin-1 homologous proteins in the venom of *Trichomalopsis sarcophagae* and *Urolepis rufipes*. These additional experiments were conducted by Qiqiang Song, who is added as a coauthor.

Following this letter are the reviewers’ comments with our responses in blue. We hope that our revised version will meet your requirements for publication in PLoS Pathogens. Thank you for your consideration!

Sincerely,

Ye Gongyin

Professor

Institute of Insect Sciences, Zhejiang University, Hangzhou 310058, China

Tel: (+86)-571-88982696; E-mail: [chu@zju.edu.cn](mailto:chu@zju.edu.cn)

Reviewer's Responses to Questions

**Part I - Summary**
Reviewer #1: This manuscript found a parasitoid serpin gene that disrupts host immunity and shows evolution of alternative splicing (AS). The AS of serpin was firstly reported in Manduca sexta by Jiang et al, 1994 JBC. It will generate up to 12 isoforms in their reactive site loops, which needs to be cited. Here, the authors reported a negative correlation between AS and gene duplication (GD) in the evolution of insect serpin genes and their associations to the parasitic life strategy. The authors also demonstrated how PpSerpin1 isoforms synergistically inhibit the host's immunity in both wasp venom and larval saliva. This is a comprehensive study encompassing evolution and function. GD is a common mechanism for generating new genes, e.g., host effector genes, but there is relatively limited research on the interplay between AS and GD. To my knowledge, this work is also the first functional analysis of a larval salivary gene in parasitoid wasps. Thus, I believe this work represents a significant advancement in understanding the evolution and function of host effector genes in parasitoid wasps.
Overall, I found the manuscript quite interesting. The analyses appear to be detailed, and the conclusions are well supported. I think the manuscript will provide a useful contribution to the field of biointeractions.

RESPONSE: We sincerely appreciate the reviewer's comments and suggestions! As suggested, we have cited Jiang et al. (1994) and Jiang et al. (1996) in both the Introduction and Discussion sections of our manuscript. We have also made revisions according to all the reviewer's suggestions below.

Reference

1) Jiang H, Wang Y, Kanost MR. Mutually exclusive exon use and reactive center diversity in insect serpins. J Biol Chem. 1994;269(1):55-8. Epub 1994/01/07. PMID: 8276850.

2) Jiang H, Wang Y, Huang Y, Mulnix AB, Kadel J, Cole K, et al. Organization of serpin gene-1 from Manduca sexta. Evolution of a family of alternate exons encoding the reactive site loop. J Biol Chem. 1996;271(45):28017-23. Epub 1996/11/08. doi: 10.1074/jbc.271.45.28017. PMID: 8910411.

Reviewer #2: The manuscript by Yan et al. describes the characterization of the contribution of alternative splicing (AS) to the functional diversity of a serine protease inhibitor (PpSerpin-1) from the venom of a parasitoid wasp (Pteromalus puparum). This work combines a plethora of methods such as genomic and phylogenetic analyses, recombinant protein expression and purification, pull-down assays and proteomics to describe an interesting biological phenomenon. I have no doubt that the extraordinary diversity of splice variants found for PpSerpin-1 was shaped by strong natural selection. However, this manuscript has several issues that should be thoroughly addressed by the authors before I can recommend its acceptance to a scientific journal. Importantly, none of these issues is glaring by itself, but altogether they justify in my opinion a major revision of the manuscript.

RESPONSE: We appreciate the reviewer's comments and evaluation! We have made revisions according to all the reviewer's suggestions below.

Reviewer #3: Although alternative splicing (AS) is considered a major source of protein diversity and evolution of gene expression in eukaryotes (the authors cite 10 reference articles), less is known about its evolution compared to gene duplication (GD) and how alternative gene splicing and gene duplication interact. This is however fundamental to assessing their respective roles in proteic diversity. The authors were original in tackling this complex issue by identifying a very good model for study (strength).
They focused on the evolution of a gene encoding a serpin (PpSerpin1) and compared it between parasitoid wasps and other insects. Using this specific model, they could demonstrate a joint role for alternative splicing and gene duplication in the level of serpins' diversity. This aspect is truly innovative and important in terms of its significance in the evolution of protein diversity mechanisms and ecological adaptation. The study also shows an exemple of how a serpin gene adapts to parasitism through alternative splicing in association with the parasitic life strategy. The knowledge of the literature is very good in terms of alternative splicing, the serpin family and parasitoid virulence factors.
I did not find any problems with the methodology or execution of either the bioinformatics or biochemical aspects. The methods are well described even if some aspects are missing for certain experiments.
For phylogeny, the authors explain that the negative correlations observed hold after phylogenetic correction. It is a pity that this correction is not better explained. It might be useful to explain further the advantages of the AS gene set and the non-AS gene set and the better conservation observed in the AS gene set. For the regulatory sequences, we have 2 Dif and Dorsal motifs enriched in the AS gene set. It would be useful to explain the link with C-term AS. PS in parasitoids compared to non parasitoids Hymenoptera and higher dN/dS values suggesting positive selection. Ppserpin1 was shown to be involved in the Toll pathway and retrieved in salivary gland and venom.

RESPONSE: We thank the reviewer for the comments and suggestions! We have made revisions to all the suggestions accordingly.

We have added more explanations about phylogenetic correction. The sentences read “To avoid biases that may arise from data non-independence due to shared evolutionary history, phylogenetic correction was performed using the independent contrasts method. Phylogenetic correction allows us to account for the evolutionary relationships among species and obtain more accurate estimates of trait correlations.”

We have further explained the advantages of the AS gene set and the non-AS gene set and the better conservation observed in the AS gene set. The sentences read “Compared to non-AS genes, AS genes are more conserved in the constitutive region but more variable in the alternative region. In addition, in the comparison of *PpSerpin-1* with its orthologous gene LOC100122505 in *N. vitripennis*, the alternative exons show significantly higher nonsynonymous substitution rates than the constitutive exons. Collectively, these results imply that AS allows for distinct regions to experience divergent evolutionary pressures, enabling rapid protein evolution in serpin RCL while preserving most of the functional backbone under strong conservation.”

For the regulatory sequences, we have further explained the link of dl and Dif transcription factors with C-terminal AS. The sentences read “In addition, the binding motifs of two transcription factors, dl and Dif, are enriched in the AS gene set compared to the non-AS gene set, particularly preferring the intron region of the coding strand of AS genes. Genes with more C-terminal AS are more likely to contain dl and Dif binding motifs. Accumulated evidence suggests that transcription factors can be directly or indirectly involved in the regulation of AS, although the mechanisms are not fully understood [53, 54]. As pre-mRNA splicing mainly takes place during transcription, extensive crosstalk has been reported between these two processes [53]. One possible explanation could be that some expanded serpin isoforms are involved in the Toll pathway. The expression of these isoforms might be regulated by Toll-pathway-related dl and Dif, probably through binding to the intron regions of serpin gene pre-mRNA.”

**Part II – Major Issues: Key Experiments Required for Acceptance**
Reviewer #1: PpSerpin1 protein was detected by Western blot in PBS incubated with P. puparum larvae and in larval saliva of P. puparum, N. vitripennis, T. sarcophagae, M. raptor, and M. uniraptor. Could the presence of PpSerpin1 protein be a result of contamination from the rupture of insect bodies? In Figure 4d and 4e, there are bands above 55 kDa, suggesting the existence of complexes formed by PpSerpin1 and its target proteases. Could these complexes be formed between PpSerpin1 and the proteases released from the parasitoid or host larvae?

RESPONSE: We thank the reviewer for pointing out this important concern! Regarding the possibility of contamination from the rupture of parasitoid larvae during the collection of saliva, we believe that the collection process is gentle and should not cause rupture of the parasitoid larvae. A video of larval saliva collection is also uploaded online https://pan.baidu.com/s/1mfmrlS4t8TRaCQnuRcu28g?pwd=k5oj. Furthermore, we conducted additional experiments to demonstrate that *P. pteromalus* larvae remained viable and showed no significant rupture after being incubated in PBS for 8 h (Fig. S5).

For the bands above 55 kDa observed in Figure 4d and 4e, we are uncertain exact reason for their presence. Here, we propose several possible explanations: a) some PpSerpin-1 isoforms may actively regulate the activity of proteases in the larval saliva; b) some PpSerpin-1 isoforms may show leaky expression in the larval saliva due to noise in AS regulation, leading to their interaction with proteases from the larval saliva itself; c) bands detected above 55 kDa are caused by non-specific binding of PpSerpin-1 antibodies.

Overall, we believe that the possibility of PpSerpin-1 in saliva being a result of contamination is minimal, and our conclusion that PpSerpin-1 does exist in larval saliva is reliable.

In the discussion section, we have also added a description of possibilities a) and b). The sentences read: “Moreover, several isoforms identified in larval saliva failed to form complexes with host hemolymph proteins. These *PpSerpin-1* isoform proteins may target proteins out of the host hemolymph, e.g., in host hemocytes or gonad cells, inside host guts, or regulate proteases from wasp larval saliva. Alternatively, the inconsistency between proteomic identification and functions of *PpSerpin-1* isoforms may be simply due to leaky expression from the noise of AS regulation [50]. More investigations are needed to test these hypotheses.”

Reviewer #2: Major comments:
1. The title is very problematic. The “parasitoid serpin gene” is a very confusing term. Also what does “showing adaptive evolution” mean? How about writing instead “A gene encoding a serine protease inhibitor from a parasitoid wasp disrupts host immunity and exhibits adaptive alternative splicing”? If this is too long, think about shorter alternatives that are less confusing and more informative than the current title.

RESPONSE: We thank the reviewer for this suggestion. We have changed the title to “A serpin gene from a parasitoid wasp disrupts host immunity and exhibits adaptive alternative splicing”.

2. While the role of PpSerpin-1 in the venom is demonstrated, it is unclear to me whether any role in the saliva of the larva is demonstrated. Is the assumption that the larva releases via saliva the serpin that inhibits host melanization? This needs to be clearly explained in the text.

RESPONSE: We apologize for the lack of clarity in the manuscript regarding the role of PpSerpin-1 in larval saliva. We conducted additional experiment to demonstrate that the larval saliva of *P. puparum* inhibits the host's melanization, and this inhibitory effect could be eliminated by PpSerpin-1 antibodies. The relevant sentences read:

“To test the function of *PpSerpin-1* in larval saliva of *P. puparum*, we demonstrated that *P. puparum* larval saliva inhibited the host's hemolymph melanization (Fig. 5a; Dunnett's test; *p* = 0.0038), and this inhibitory effect could be eliminated by the antibody of PpSerpin-1 (Fig. 5a; Dunnett's test; *p* > 0.05).”

3. Lines 253-271: it is unclear how the proteomic analysis contributed to the understanding of the role of the splice variants that do not cause melanization. Please explain.

RESPONSE: We thank the reviewer for this suggestion. We have added a paragraph to address this and the next concerns. The paragraph reads: “Not all isoforms recruited into wasp venom or larval saliva inhibit host melanization. These isoforms may serve other functions, such as regulating the host's Toll pathway or modulating host metabolism. Proteomic identification results from the pull-down assays of these isoforms can provide hints into their targets and functions, which will be an interesting future direction. Moreover, several isoforms identified in larval saliva failed to form complexes with host hemolymph proteins. These *PpSerpin-1* isoform proteins may target proteins out of the host hemolymph, e.g., in host hemocytes or gonad cells, inside host guts, or regulate proteases from wasp larval saliva. Alternatively, the inconsistency between proteomic identification and functions of *PpSerpin-1* isoforms may be simply due to leaky expression from the noise of AS regulation [50]. More investigations are needed to test these hypotheses.”

4. In many genes not all splice variants are functional because AS is a somewhat stochastic process that introduces some “noise”. This possibility should be mentioned in the discussion. Please see Wan and Larson 2018 Genome Biology 19: 86.

RESPONSE: We appreciate the reviewer's suggestion to mention the possibility of non-functional AS as a result of noise in AS regulation. We also thank the reviewer for providing us with the relevant reference, Wan and Larson 2018 Genome Biology 19: 86. In light of this, we have added a discussion to address this aspect and have cited the suggested reference. A paragraph has been added to address this and the previous concerns. The paragraph reads: “Not all isoforms recruited into wasp venom or larval saliva inhibit host melanization. These isoforms may serve other functions, such as regulating the host's Toll pathway or modulating host metabolism. Proteomic identification results from the pull-down assays of these isoforms can provide hints into their targets and functions, which will be an interesting future direction. Moreover, several isoforms identified in larval saliva failed to form complexes with host hemolymph proteins. These *PpSerpin-1* isoform proteins may target proteins out of the host hemolymph, e.g., in host hemocytes or gonad cells, inside host guts, or regulate proteases from wasp larval saliva. Alternatively, the inconsistency between proteomic identification and functions of *PpSerpin-1* isoforms may be simply due to leaky expression from the noise of AS regulation [50]. More investigations are needed to test these hypotheses.”

5. It seems that the proteomics raw data was not submitted to a public database. The LC-MS/MS data must be submitted to a public database such as PRIDE by EMBL-EBI (see https://www.ebi.ac.uk/pride/) and be publicly available.

RESPONSE: We thank the reviewer for pointing out this issue. We submitted raw proteomics data to the PRIDE database (<https://www.ebi.ac.uk/pride/>) and provided accession numbers in the “Data availability” section. “The accession numbers of the raw proteomics data are PXD044136 and PXD044199 in PRIDE (Proteomics IDEtification Database).”

Reviewer #3: i) In computer analyses (here, phylogeny), it would be a good idea to include a paragraph explaining why it is important to make a phylogenetic correction (avoid bias) and detailing the methodology used to do so.

RESPONSE: We appreciate the reviewer's suggestion to include a paragraph explaining the importance of making a phylogenetic correction and detailing the methodology used. We have added sentences to address this concern. The paragraph reads “To avoid biases that may arise from data non-independence due to shared evolutionary history, phylogenetic correction was performed using the independent contrasts method. Phylogenetic correction allows us to account for the evolutionary relationships among species and obtain more accurate estimates of trait correlations. For independent contrasts, the phylogenetic species tree was generated by phyloT v2 (<https://phylot.biobyte.de/>). The branch length was estimated by compute.brlen in the R package “ape” v5.6.2 [82] using Grafen's (1989) methods [83]. Independent contrasts were conducted using the “pic” function in the R package “ape” [82]. Correlations through origins were estimated for independent contrasts using the R package “picante” v1.8.2 [84].”

ii) I think it would be interesting to go into more detail in the motif scanning approach in the manuscript, including the precise methodology which is not yet available.

RESPONSE: We have provided a more detailed description of the methods used for motif scanning. The sentence reads “For binding motifs of dl (M02712_2.00) and Dif (M03953_2.00), hits were detected with a *p* value threshold of 1 using FIMO in MEME [77].”

iii) The most conclusive experiment is clearly based on the production of PpSerpin 1 isoforms (constitutive fragments without signal peptides), a production that makes it possible to test their capacity to inhibit host phenoloxidase activity, including a dose-dependent aspect. It would have been interesting in this context to test the association of some of these isoforms. The pull-down assay experiments carried out to identify potential interacting targets could have been extended to more isoforms, but it would have been time-consuming and the results are already very conclusive with the identification of target host serine proteases and complexes.

RESPONSE: We appreciate the reviewer's helpful suggestions. Indeed, we expressed recombinant proteins for all 21 identified isoforms and tested the inhibitory capacity of all these isoforms against host melanization. For pull-down, we only conducted assays for 4 isoforms that showed inhibitory activity and 9 other isoforms identified in the venom or larval saliva. We acknowledge that due to potential identification omissions in the venom and larval proteome, there might be other isoforms that could interact with the host. Thus, performing pull-down assays on more isoforms will be helpful. However, this would also be time-consuming and could delay the publication of the paper. As the reviewer pointed out, “the results are already very conclusive with the identification of target host serine proteases and complexes.” We are grateful for the reviewer's understanding, and we will consider extending our investigations to more isoforms in future studies.

**Part III – Minor Issues: Editorial and Data Presentation Modifications**
Reviewer #1: (1) As shown in Fig. 4e, the presence of the PpSerpin1 protein seems to be widespread in the larval saliva of P. puparum and its relatives. Accordingly, it is worth investigating whether the PpSerpin1 protein is also found in the venom of these parasitoid species. This can be explored by Western blotting utilizing PpSerpin1 antibodies. Furthermore, several published articles have addressed the identification of venom proteins in Nasonia and its relatives. For instance, Martinson et al. (2017) reported the venom proteome of N. vitripennis, N. giraulti, T. sarcophagae, and U. rufipes. It would be interesting to determine if the PpSerpin1 protein is also present in the venom proteomic data reported in these studies.
Reference: Martinson EO, Mrinalini, Kelkar YD, Chang CH, Werren JH. The evolution of venom by co-option of single-copy genes. Curr Biol. 2017;27(13):2007-13.

RESPONSE: We thank the reviewer for this suggestion! We have conducted further research and examined previously published literature. By Western blotting, we detected PpSerpin-1 in the venoms of *P. puparum* and its relative species *N. vitripennis*, *Muscidifurax raptor*, and *M. uniraptor*. By examining published venom sequences, we also identified PpSerpin-1 homologous proteins in the venom of *Trichomalopsis sarcophagae* and *Urolepis rufipes*. A new paragraph has been added to our manuscript.

“In our previous study, PpSerpin-1O was isolated from *P. puparum* venom [22]. RNA-seq data showed that multiple *PpSerpin-1* isoforms are expressed in the venom gland (Fig. 4c). We also re-analyze the published *P. puparum* venom proteomic data [37] and identified isoforms B, G, O and P. By Western blotting, PpSerpin-1 and its homologous proteins were detected in the venoms of *P. puparum* and its relative species *N. vitripennis*, *Muscidifurax raptor*, and *M. uniraptor* (Fig. 4d). Additionally, by examining published venom sequences, we also identified PpSerpin-1 homologous proteins in the venom of *Trichomalopsis sarcophagae* and *Urolepis rufipes*, exhibiting 90.1% and 91.6% identity to the constitutive sequence of PpSerpin-1 protein, respectively [17, 35].”

(2) The methods used for immune simulation must be clearly described. A related question: Is there any overlap between the isoforms induced by immune stimulation and the isoforms recruited to venom or larval saliva? Is there a correlation between the expression levels of isoforms in the venom gland or larval salivary gland and the fold changes after immune simulation? Are the isoforms that are upregulated by immune stimulation more likely to be recruited into venom or larval saliva?

RESPONSE: We thank the reviewer for the suggestions! We have added a paragraph of “Microbial stimulus of P. puparum” in the methods section. The paragraph reads:

“**Microbial stimulus of *P. puparum***

Freshly cultured *M. luteus*, *E. coli*, and *B. bassiana* were harvested by centrifugation and subsequently washed with sterile PBS three times. These microbes were suspended in PBS at a density of 5×10^7^ cells/mL. Two-day old *P. puparum* female adults were selected and anesthetized using carbon dioxide. A sterilized acupuncture needle was immersed in the suspension containing either bacteria or fungi and then inserted into the inter-segmental space of the parasitoid wasp's abdomen. Wasps that were pricked with sterile PBS were used as a control group. Female wasps were then collected at 6, 24, and 48 h after the microbial stimulus.”

In addition, we tested both Pearson and Spearman correlations between the expression levels of isoforms in the venom gland or larval salivary gland and the fold changes after immune simulation, and we found no significant correlations (all tests: *p* >0.05). We also tested whether isoforms detected in larval saliva showed a higher fold change after immune stimulation than those not detected in saliva. No significant differences were detected (Mann‒Whitney U test; all comparisons: *p* > 0.05). Similar results were observed for venom isoforms.

(3) The authors compared several features between parasitoid wasps and non-parasitoid insects. In addition to parasitoid wasps, are there any other parasitic insects included in the analyzed dataset? If so, do the findings regarding parasitoid wasps also apply to these other parasitic insects?

RESPONSE: We examined the dataset used in our study and found no other parasitic insects besides parasitoid wasps. We are also interested in whether similar phenomena exist among various parasitic insects, and future expansion of the dataset may provide clues in this regard. This will be a direction for our future research.

(4) For the protein structure prediction, it is necessary to provide more details and parameters, including whether templates were used in AlphaFold2 and the confidence level of predicted models, such as pLDDT values.

RESPONSE: We thank the reviewer for pointing out this issue. We have provided more details and parameters for AlphaFold2 prediction. The sentences read: “The protein structure of PpSerpin-1F was modeled using AlphaFold2 with default settings [62, 63]. No structural template was used. All five AlphaFold2 models were tested. Model 4 was selected as the best prediction based on its highest pLDDT score of 88.7.”

(5) In abstract, “…have been recruited to both wasp venom and larval saliva”. Again, is there any evidence, such as Wb or proteomics (apart from Fig 4c), to show that Ppserpin in the venom? Probably it is related to the previous publication.

RESPONSE: We apologize for the lack of clarity in the manuscript regarding the presence of PpSerpin-1 in wasp venom. We have conducted further research and examined previously published literature. By Western blot, we detected PpSerpin-1 in the venoms of *P. puparum* and its relative species *N. vitripennis*, *Muscidifurax raptor*, and *M. uniraptor*. By examining published venom sequences, we also identified PpSerpin-1 homologous proteins in the venom of *Trichomalopsis sarcophagae* and *Urolepis rufipes*. A new paragraph has been added to our manuscript.

“In our previous study, PpSerpin-1O was isolated from *P. puparum* venom [22]. RNA-seq data showed that multiple *PpSerpin-1* isoforms are expressed in the venom gland (Fig. 4c). We also re-analyze the published *P. puparum* venom proteomic data [37] and identified isoforms B, G, O and P. By Western blotting, PpSerpin-1 and its homologous proteins were detected in the venoms of *P. puparum* and its relative species *N. vitripennis*, *Muscidifurax raptor*, and *M. uniraptor* (Fig. 4d). Additionally, by examining published venom sequences, we also identified PpSerpin-1 homologous proteins in the venom of *Trichomalopsis sarcophagae* and *Urolepis rufipes*, exhibiting 90.1% and 91.6% identity to the constitutive sequence of PpSerpin-1 protein, respectively [17, 35].”

(6) In Figure 6, “Pp” in “PpSerpin-1” is italicized, while “Serpin-1” is in regular font. Similarly, “Pr” in “PrPAP1” is italicized, while “PAP1” is in regular font. Generally, gene names should be italicized, while protein names should not be. It is important to ensure consistency throughout the entire document in adhering to these conventions.

RESPONSE: We thank the reviewer for pointing out this issue. We have unified the gene and protein names throughout the text, figures, and supplementary materials.

(7) The clarity of Fig 1a and 1c needs to be improved. The lines in the figures are not clear, which hinders readability.

RESPONSE: We have improved the clarity of both Fig 1a and Fig 1c in the revised manuscript.

(8) In line 37, “Pteromalus puparum” should be italicized.

RESPONSE: Corrected.

(9) In line 91, change “alternate” to “alternative”

RESPONSE: Corrected.

(10) In line 913, change “Western blot detection in larval saliva of Nasonia vitripennis, Trichomalopsis sarcophagae, Muscidifurax raptor and M. uniraptor using PpSerpin1 antibodies.” to “Western blot detection in larval saliva of P. puparum, Nasonia vitripennis, Trichomalopsis sarcophagae, Muscidifurax raptor, and M. uniraptor using PpSerpin1 antibodies.”

RESPONSE: Corrected.

Reviewer #2: Minor comments:
1. Lines 47-48: “ubiquitous” is a confusing term in the context of this sentence. Please change to “frequent”.

RESPONSE: Corrected.

2. Lines 221-223: the term “accelerated protein evolution” is a bit outdated. Please use the term “positive selection diversifying protein sequence” instead.

RESPONSE: Corrected.

3. dN and dS should be italicized.

RESPONSE: Corrected.

4. Lines 230-231: this suggestion is highly speculative and should be moved to the discussion. Furthermore, it should be emphasized this is based on data from Drosophila and not from wasps.

RESPONSE: We thank the reviewer for pointing out this issue. We have moved this speculation to the discussion and emphasized that this speculation is based on Drosophila data. “Several PpSerpin-1 isoforms can be upregulated by *M. luteus* and *B. bassiana*, suggesting that PpSerpin-1 may be involved in the wasp’s Toll pathway, which is preferentially activated by gram-positive bacteria and fungi in *Drosophila* [45].”

5. Line 517: gastropod is a mollusk. I think you mean another term?

RESPONSE: Corrected. The sentence now reads “Plasma was harvested by cutting the hind legs of *P. rapae* larvae with scissors and diluted four times into ice-cold TBS buffer (20 mM Tris, 150 mM NaCl, pH=7.6).”

6. Lines 907-908: the figure legend of panel 4b should be more detailed. I guess this heatmap represents RNA-Seq results? This should be clarified.

RESPONSE: We appreciate the reviewer’s feedback and have updated the figure legend of panel 4b. The heatmap indeed represents the RNA-Seq results, and we have clarified this in the legend. The revised sentence reads: “Expression levels were calculated from RNA-seq data.”

7. Lines 919-920: why are there two bands for some of the recombinant proteins? Is this incomplete denaturation or some truncated by-product?

RESPONSE: We thank the reviewer for pointing out this phenomenon. The presence of by-products could affect the precise quantification of the active protein, but it will not impact the main conclusions drawn in the manuscript. We apologize that we do not know the exact cause of this phenomenon. We speculate that the presence of two bands for some of the recombinant serpin proteins might be attributed to the occurrence of truncated by-products during the expression process. We have added a note in the figure legend of Fig. S6. “Some isoform proteins show two bands, which might be truncated by-products during the expression process.”

Reviewer #3: i) In some places in the manuscript, certain words are misspelt (letters not in the right order) and should be corrected.

RESPONSE: We thank the reviewer for the careful reading and pointing out this issue. We have carefully reviewed the entire manuscript and corrected the spelling errors accordingly.

ii) I find that the figures are a bit crowded (number of graphs per figure) and I suggest simplifying them where possible and desirable.

RESPONSE: We thank the reviewer for this suggestion. We have simplified Figure 5 and moved some data into the supplementary file.
